# Supramolecular block copolymers by kinetically controlled co-self-assembly of planar and core-twisted perylene bisimides

**DOI:** 10.1038/ncomms8009

**Published:** 2015-05-11

**Authors:** Daniel Görl, Xin Zhang, Vladimir Stepanenko, Frank Würthner

**Affiliations:** 1 Institut für Organische Chemie and Center for Nanosystems Chemistry, Universität Würzburg, Am Hubland, Würzburg 97074, Germany

## Abstract

New synthetic methodologies for the formation of block copolymers have revolutionized polymer science within the last two decades. However, the formation of supramolecular block copolymers composed of alternating sequences of larger block segments has not been realized yet. Here we show by transmission electron microscopy (TEM), 2D NMR and optical spectroscopy that two different perylene bisimide dyes bearing either a flat (A) or a twisted (B) core self-assemble in water into supramolecular block copolymers with an alternating sequence of (A_m_BB)_n_. The highly defined ultralong nanowire structure of these supramolecular copolymers is entirely different from those formed upon self-assembly of the individual counterparts, that is, stiff nanorods (A) and irregular nanoworms (B), respectively. Our studies further reveal that the as-formed supramolecular block copolymer constitutes a kinetic self-assembly product that transforms into thermodynamically more stable self-sorted homopolymers upon heating.

## Abstract (web summary)

Block co-polymers are becoming of ever-increasing importance in polymer science. Here, the authors show that the co-assembly of two perylene bisimide dyes leads to supramolecular block copolymer structures with a regular alternating sequence of short blocks of respective homoaggregates.

## Graphical abstract

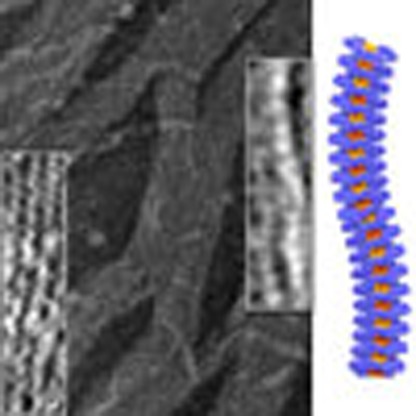

Self-assembly has become a key technology for the creation of nanostructured materials with desirable properties for application, for example, in organic electronics, molecular sensing and bionanotechnology^1^,^2^,^3^. However, major focus in this field being so far on single-component self-assembly to elucidate structure–property relationships. In contrast, multicomponent assembly is by far less explored, despite its high promises for the construction of novel nanosized composite materials. With his pioneering work on the elucidation of mixtures of established small-sized self-assembling systems, in particular based on hydrogen-bonding-recognition patterns, Isaacs established the research field of self-sorting phenomena^4^ that has gained increasingly more importance during the last decade. In this seminal work as well as in most of the subsequent contributions, however, typically only small-sized finite and analytically assignable supramolecular architectures are addressed^5^,^6^,^7^,^8^,^9^.

On the other hand, for large^10^,^11^ and non-finite self-assembled systems^12^,^13^,^14^ few studies on multicomponent self-assembly with detailed insights into the self-assembly sequence exist. Remarkably, perfect self-sorting into A_n_B_m_ copolymers has been afforded for unidirectionally growing systems by sequential assembly^15^,^16^,^17^,^18^,^19^, while perfect mixing into supramolecular copolymers with alternating sequences (AB)_n_ has been realized by well-directed molecular design for mutual interactions of the individual components^20^,^21^,^22^,^23^,^24^,^25^. However, unravelling constitutional aspects of supramolecular polymer architectures formed by bottom-up self-assembly of multicomponent mixtures remains still challenging, particularly in water. One reason for the reluctance of researchers to enter this field is given by the difficulties encountered in the analysis of the large-sized and polydisperse self-assembly products. Another reason might be the additional challenges encountered by pathway complexity that arises when the growth of a larger nano- or even micrometre-sized structure operates either under kinetic or thermodynamic control, depending on the experimental conditions^26^,^27^,^28^. As pointed out by Rybtchinski and by Fernández in two recent reviews, pathways towards kinetic products are in particular to be considered for the self-assembly of larger aromatic π-systems in water because of the hydrophobic effect^29^,^30^. Our previous achievements^31^ in elucidation of the hierarchical growth of a planar perylene bisimide (PBI) amphiphile (PBI **1**) in water into nanorods and nanolamellae by transmission electron microscopy (TEM) encouraged us to elucidate the co-assembly of planar PBI **1** with core-twisted and conformationally more flexible (‘soft') PBI amphiphile **2**.

Here we report our unique findings on the bicomponent system PBI **1**/PBI **2** that hydrophobic interactions lead to the formation of kinetically controlled supramolecular copolymers in water as explored by TEM and two-dimensional (2D) nuclear magnetic resonance (NMR) studies as well as optical spectroscopy. These results provide novel insights into the evolution of π-stacked supramolecular structures in aqueous media^32^,^33^,^34^,^35^,^36^.

## Results

### Molecular design

**Figure 1:**
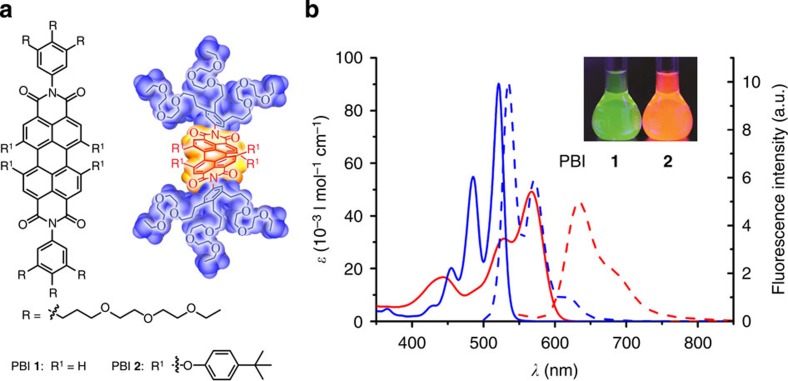
Molecular design and optical properties of PBIs 1 and 2. (**a**) Chemical structures and space-filling (CPK) model of PBIs **1** and **2**. (**b**) Ultraviolet–visible absorption spectra (solid lines) and corresponding fluorescence spectra (dashed lines) of PBI **1** (blue) and PBI **2** (red) in THF; concentration, ∼1 × 10^−5^ M of each sample. Excitation wavelength is 470 nm. Inset, photograph of PBI **1** and PBI **2** solutions in THF under ultraviolet light.

The molecular structures of amphiphilic PBI **1** (ref. 31) and PBI **2** are shown in Fig. 1a and the synthesis of the new PBI **2**, along with its characterization data (Supplementary Fig. 1), is reported in the Supplementary Methods. The perylene core of PBI **1** is almost flat, while that of PBI **2** is enforced to twist by ∼30° due to the presence of four bulky aryloxy substituents in the bay region^37^,^38^,^39^. These bay substituents also strongly influence the optical properties of PBIs (Fig. 1b). Thus, core-twisted PBI **2** molecularly dissolved in tetrahydrofuran (THF) displays a 46-nm red-shifted absorption maximum compared with that of planar PBI **1** and a second absorption band at a shorter wavelength of 444 nm, which can be attributed to a partially symmetry allowed S_0_–S_2_ transition with a transition dipole moment perpendicular to the molecular long axis^40^. Consequently, PBI **1** shows a green fluorescence, while PBI **2** a red one.

### Self-assembly of unimolecular PBI systems

**Figure 2:**
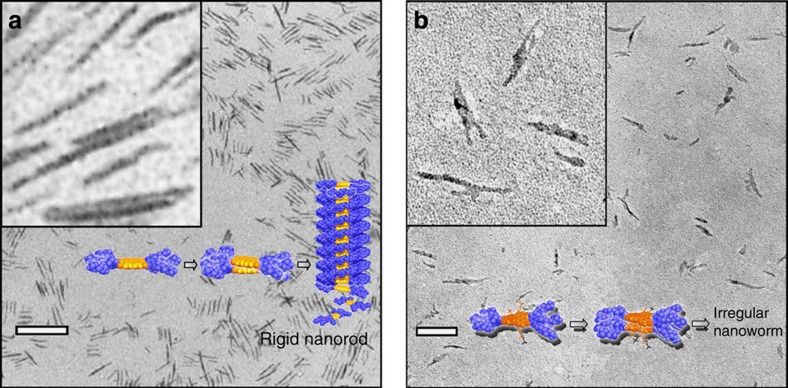
Morphology of unimolecular PBI self-assemblies. (**a**) TEM image of PBI **1** aggregates in water, [PBI **1**]=0.05 mM (0.077 mg ml^−1^) and schematic space-filling models illustrating the self-assembly of PBI **1**. Scale bar, 100 nm. Inset, magnified TEM image of the same sample. (**b**) TEM image of PBI **2** aggregates in water, [PBI **2**]=0.03 mM (0.077 mg ml^−1^) and schematic space-filling models illustrating the self-assembly of PBI **2**. Scale bar, 50 nm. Inset, magnified TEM image of the same sample.

We have first studied the self-assembly behaviour of the new PBI **2** in aqueous medium and compared it with that of the previously investigated PBI **1**. For PBI **1**, we had observed a very slow (ca. 2 days) dissolution process in water, leading to either one-dimensional (1D) nanorods (at a concentration of ∼0.05 mM) or nanolamellae (at concentrations >0.1 mM)^31^. Because the dissolution process is such slow and the conversion between nanorods and nanolamellae proved to be reversible, it is reasonable to consider that these aggregate structures are thermodynamically equilibrated. This view is further supported by the fact that these structures are formed pathway independently. Thus, the same structures are also generated upon injection of water to molecularly dissolved PBI **1** in THF and subsequent evaporation of THF ensuring a slow gradual increase of water content in the solvent mixture. Different from PBI **1**, PBI **2** does not dissolve in pure water under ambient conditions due to its increased hydrophobicity imparted by the aryloxy substituents at bay area. However, a stable suspension of aggregates was obtained by adding water to molecularly dissolved PBI **2** in THF and subsequent slow evaporation of THF from the THF/H_2_O solution of PBI **2**. Very different from the homogeneous and rigid segmented nanorod structures observed for PBI **1** (Fig. 2a), TEM analysis of the aggregates of PBI **2** revealed more irregular and flexible worm-like aggregates at low concentration of 3 × 10^−5^ M in water (Fig. 2b and Supplementary Fig. 2). The formation of these nanoworm structures is apparently evoked by the twisting of the perylene core of PBI **2**, leading to a different and less regular packing arrangement compared with PBI **1** aggregates.

**Figure 3:**
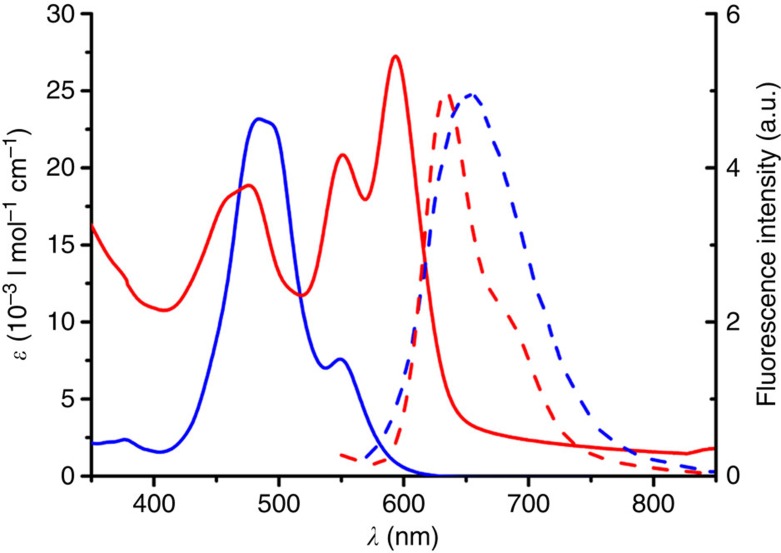
Optical properties of unimolecular PBI self-assemblies. Ultraviolet–visible absorption spectra (solid lines) and corresponding normalized fluorescence spectra (dashed lines) of PBI **1** (blue) and PBI **2** (red) in water; concentration, ∼1 × 10^−5^ M of each sample. Excitation wavelength is 470 nm. Magic-angle setup was applied.

To understand the formation of these nanoworms upon removal of THF from THF/H_2_O mixtures, we first performed concentration-dependent ultraviolet–visible studies of PBI **2** in various THF/H_2_O mixtures. These studies reveal a transition from monomeric to aggregated species upon increasing concentration, which is accompanied by a decrease and a small bathochromic shift (<5 nm) of the absorption maximum (Supplementary Fig. 3). The corresponding plot of extinction versus concentration of PBI **2** could be fitted best to the dimer model^41^ (see Supplementary Fig. 3, insets). This is reasonable because the bulky bay substituents prevent the π-surfaces in PBI **2** dimer from further stacking interactions as already described in our earlier work on the aggregation of related core-distorted PBIs in organic solvents^37^. Upon increasing the water content in THF/H_2_O mixtures from 7:3 to 5:5 (v/v) the binding constant *K* increases only slightly from *K*(PBI **2**)=5 × 10^1^ M^−1^ (7:3) to *K*(PBI **2**)=4 × 10^3^ M^−1^ (5:5), but then distinctly to *K*(PBI **2**)=7 × 10^4^ M^−1^ when reaching a specific solvent composition of THF/H_2_O=4:6. A similar transition from monomers to dimers is also observed upon increasing the water content and concomitantly the hydrophobic effect in dilute solutions of PBI **2** in THF/H_2_O mixtures (Supplementary Fig. 4). Driven by the hydrophobic effect, these PBI **2** dimers are then further aggregated into structurally less-defined nanoworms in pure water as revealed by our TEM studies. The ultraviolet–visible spectrum of PBI **2** in pure water (Fig. 3) shows a more pronounced bathochromic shift (27 nm) compared with those in the THF/H_2_O mixtures as well as some scattering effects (baseline does not go to zero), pinpointing the suspension character of the sample.

The binding constants for PBI **1** self-assembly in THF/H_2_O mixtures were determined by the isodesmic model that assumes equal binding constants for both PBI π-surfaces in accordance with our earlier work^42^ and the observation of defined 1D aggregates by TEM (Fig. 2). In contrast to PBI **2**, a pronounced hypsochromic shift of the absorption maximum is observed upon aggregation of PBI **1** (Supplementary Fig. 5). As expected, the binding constant of PBI **1** increases with increasing amount of water from *K*(PBI **1**)=1 × 10^3^ M^−1^ in THF/H_2_O 7:3 to *K*(PBI **1**)=4 × 10^4^ M^−1^ in THF/H_2_O 4:6. Interestingly, for higher THF contents (>50%) PBI **1** exhibits significantly higher binding constants than PBI **2** whilst for H_2_O-enriched mixtures, the dimerization constant for PBI **2** surpasses the aggregation constant of PBI **1** due to the pronounced hydrophobicity of PBI **2** (Supplementary Fig. 6). Unfortunately, for more water-enriched mixtures (>60% H_2_O), binding constants for both PBI **1** and PBI **2** could not be determined reliably due to very strong aggregation that prohibits analysis of concentration-dependent ultraviolet–visible experiments.

As shown in Fig. 3, the aggregates of PBI **1** and PBI **2** in water exhibit indeed very different ultraviolet–visible spectra. For PBI **1** the major band is hypsochromically displaced compared with that of the monomers (in THF), while for PBI **2** a bathochromic shift compared with the monomer band is observed. Moreover, owing to pronounced excitonic coupling between the closely stacked dyes^43^, spectra of PBI **1** aggregate do not show the vibronic coupling pattern of the monomeric dyes. However, such vibronic transitions are clearly observed for the aggregates of PBI **2** as expected for less-tightly stacked dyes owing to the twisted core and the bulky phenoxy substituents. Compared with the PBI **1** aggregates, also quite different fluorescence bands are observed for PBI **2** aggregates (Fig. 3 and Supplementary Fig. 7). The former display typical excimer-type broad emission band^44^ with a large Stokes shift (emission maximum at 650 nm), whereas the fluorescence spectra of the latter show only a moderate Stokes shift and have similar shapes as the fluorescence bands of the molecularly dissolved dyes. These features suggest that the core-twisted PBI dyes **2** are only weakly coupled in aggregates, which corroborates our earlier conclusions that the main driving force for self-assembly of core-twisted PBI **2** is the hydrophobic effect whilst additional strong π–π interactions are operative in aggregates of planar PBI **1**.

### Co-assembly of PBI 1 and PBI 2

**Figure 4:**
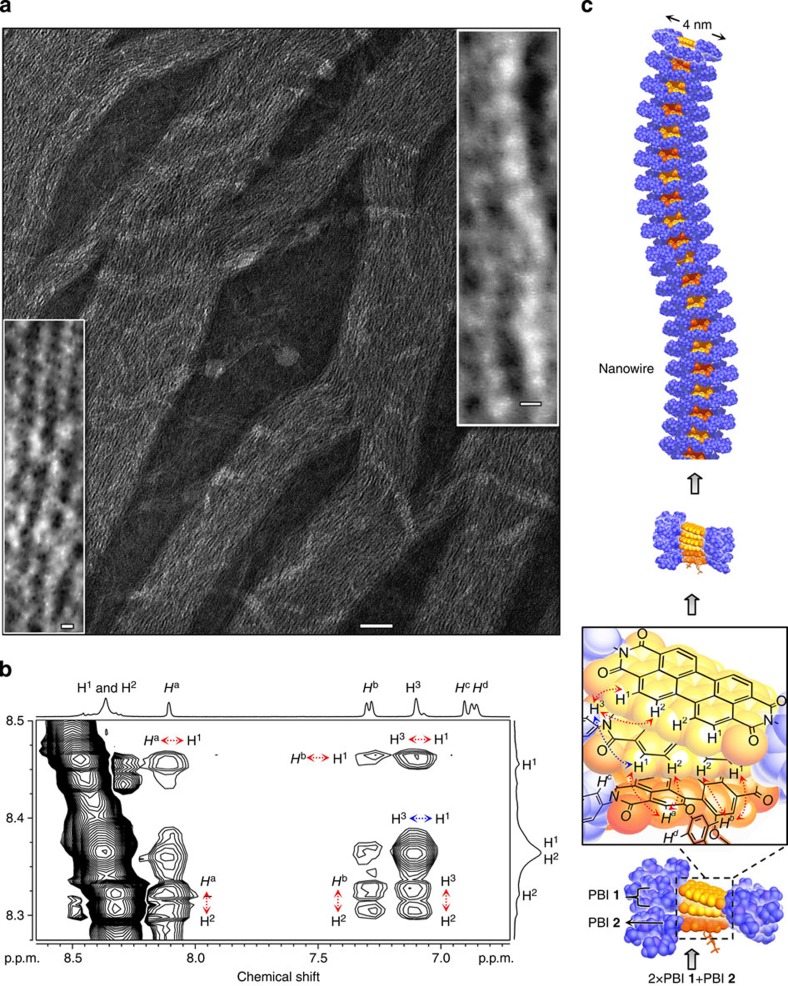
Co-assembly of planar and core-twisted PBIs. (**a**) TEM images of segmented nanowires formed by co-assembly of PBI **1**/PBI **2** in water. Scale bar, 50 nm. Inset, magnified TEM images of the single-molecule-thin nanowires. Scale bars, 2 nm. [PBI **1**]:[PBI **2**]=2:1 in molar ratio, [PBI **1**]=3.2 × 10^−4^ M (0.5 mg ml^−1^). (**b**) ^1^H, ^1^H-ROESY spectroscopy for PBI **1**/PBI **2** co-assemblies in D_2_O/[D_8_]THF (400/600 μl). [PBI **1**]:[PBI **2**]=2:1, [PBI **1**]=4.9 × 10^−3^ M. (**c**) Schematic illustration based on space-filling model for PBI **1**/PBI **2** co-assembly. For clarity, only representative protons are shown in the structural model of one co-assembling segment from semiempirical AM1 calculations.

Since both types of aggregates, namely well-dissolved PBI **1** nanorods and suspension-forming PBI **2** nanoworms, respectively, can be generated by the same preparation method, that is, by injection of water to the respective PBI dye monomer solution in THF and subsequent slow removal of THF under ambient conditions, we were able to address the effect of water on the self-assembly of a mixture of both PBI dyes and to elucidate the structural features of the aggregates formed. Thus, we prepared a solution of a 2:1 mixture of PBI **1** and PBI **2** in THF, injected water to this solution and slowly removed the organic solvent under ambient conditions. Microscopic analyses by TEM (Fig. 4 and Supplementary Fig. 8) and atomic force microscopy (Supplementary Fig. 9) revealed that the assemblies of this mixed system in water are quite different from those of the individual components, as the co-assemblies grow along 1D axis into long-range, structurally ordered nanowires. These nanowires are ultrathin with uniform diameter of 3–4 nm (Fig. 4), and are among the thinnest organic nanowires ever revealed by electron microscopy^45^,^46^. These ‘single-molecule'-thin nanowires are also extraordinarily long up to 5 μm and display high aspect ratio. Segmented structures within the nanowires could be resolved by TEM (Fig. 4, inset). Cryo-TEM images (Supplementary Fig. 10) further corroborate their 1D constitution, which significantly differs from previously reported PBI **1** nanorod and nanolamellae self-assemblies^31^, as well as from those of PBI **2** (Fig. 2).

The different nanostructures observed by TEM for pure PBI **1**, pure PBI **2** and PBI **1**/PBI **2** mixtures are a strong indication for a co-assembly process between PBI **1** and PBI **2** molecules. However, on this macroscopic level, we cannot get insight into the local contacts and distinguish between, for example, alternating (AB)_n_- and block A_n_B_m_-type co-aggregates. Accordingly, the initial steps for the formation of supramolecular structures of this PBI **1**/PBI **2** mixed system were further analysed by NMR spectroscopy. For this purpose, D_2_O was gradually added to a solution of PBI **1** and PBI **2** in a 2:1 molar ratio in deuterated THF and the chemical shifts of the perylene protons were monitored by NMR spectroscopy (Supplementary Fig. 11). With increasing D_2_O content, the chemical shifts of the perylene protons of both planar PBI **1** (H^1^ and H^2^) and core-twisted PBI **2** (H^a^ and H^b^) move to higher field as a consequence of π–π stacking induced by water (D_2_O) addition. Two-dimensional ^1^H–^1^H-ROESY NMR spectroscopy was used to elucidate three-dimensional structures and conformations of PBI **1**/PBI **2** mixtures by through-space proton correlations. In ROESY NMR spectra (Fig. 4b) of 2:1 mixture of **1** and **2** cross-peaks appear between H^a^ (8.10 p.p.m.) and H^1^ (8.45 p.p.m.), H^a^ (8.10 p.p.m.) and H^2^ (8.32 p.p.m.), H^b^ (7.28 p.p.m.) and H^1^ (8.45 p.p.m.), and H^b^ (7.28 p.p.m.) and H^2^ (8.32 p.p.m.). This suggests that the protons H^1^ and H^2^ of PBI **1** have through-space correlation with the protons H^a^ and H^b^ of PBI **2**, implying an intermolecular face-to-face co-assembly of planar and core-twisted PBI molecules. In addition, the cross-peaks between the protons of the same kind of molecules, particularly PBI **1**, appear between H^3^ (7.10 p.p.m.) and H^1^ (8.45 p.p.m.), and H^3^ (7.10 p.p.m.) and H^2^ (8.32 p.p.m.). To distinguish the intramolecular (through-bond, shown in blue arrow) and intermolecular (through-space, shown in red arrow) correlation signals, we compared 2D ^1^H–^1^H TOCSY (total correlated spectroscopy) NMR spectra, which only show the through-bond cross-peak signals (Supplementary Figs 12 and 13). The cross-peak between H^3^ (7.10 p.p.m.) and H^1^ (8.36 p.p.m.) observed in ROESY spectra also appear in ^1^H–^1^H TOCSY spectra, which confirms that this cross-peak signal arose from the intramolecular (through-bond) correlation. Accordingly, this NMR analysis provided evidence for the evolvement of mixed dye assemblies, that is, contact of π-surfaces between PBI **1** and PBI **2**, during the formation process of the nanowires in THF/water mixtures.

**Figure 5:**
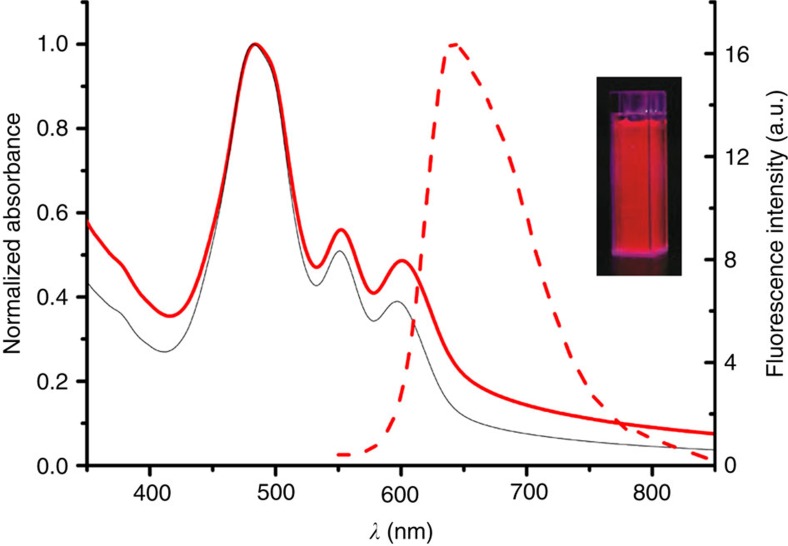
Optical properties of PBI 1/PBI 2 nanowires. Normalized absorption spectrum (red solid) and corresponding fluorescence spectrum (red dashed line, *λ*_exc_=480 nm, magic-angle setup) of nanowires formed by co-assembly of PBI **1**/PBI **2** in a molar ratio of 2:1 ([PBI]=7.5 × 10^−5^ M in water) and calculated absorption spectrum (black solid) for a 2:1 mixture of PBI **1** and PBI **2** based on the linear superposition of normalized absorption spectra of PBI **1** and PBI **2** (see Fig. 3), respectively, in water. Inset, photograph of a solution of nanowires in water under ultraviolet light.

The nanowires of PBI **1**/PBI **2** (2:1) mixed system were further analysed by optical spectroscopy. The absorption spectrum (Fig. 5) shows three maxima (at 483, 552 and 601 nm) and is again affected by scattering effects. Since both molecules PBI **1** (ref. 31) and PBI **2** do not show concentration-dependent absorption changes in water, we compared the spectrum of the mixed system with a calculated spectrum based on the spectra of the single components in water. This comparison reveals same spectral characteristics, suggesting that the mixed PBI **1**/PBI **2** system is composed of blocks of PBI **1** aggregates, exhibiting same optical properties like the previously reported PBI **1** aggregates, and blocks of PBI **2** aggregates. Accordingly, this result suggests that the PBI **1** and PBI **2** molecules are not alternately stacked in (AB)_n_ co-assemblies, which would exhibit different spectral properties. This outcome is in apparent contradiction with the previous insights gained by NMR spectroscopy. However, both findings can be reconciled in a structural model of these nanowires based on blocks of short homoaggregates that are dominated by the excitonic coupling pattern arising from same neighbour molecules, which further assemble into the ultrathin nanowires. Indeed, as revealed from our earlier work^37^, PBI **2** has a strong preference for dimerization, but is reluctant to grow into more extended supramolecular polymers (anti-cooperative self-assembly) and, accordingly, dimeric blocks of PBI **2** constitute a very reasonable option for the size of the ‘soft' block in these mixed aggregate nanowires.

To assess the effect of the molar ratio of PBI **1** and PBI **2** on the co-assemblies of these PBIs and thus on the optical properties of the resulted aggregates, we have investigated the mixtures of these PBIs in 4:1, 8:1 and 10:1 molar ratios in water by ultraviolet–visible absorption spectroscopy. The absorption profiles of these mixtures were elucidated similarly as for the 2:1 mixture shown in Fig. 5 and are displayed in Supplementary Fig. 14. The spectra of the various mixtures are all in good agreement with the calculated ones, suggesting that the mixed aggregates are in all cases composed of individual self-assembled PBI **1** and PBI **2** blocks, and thus exhibit the same optical properties as the respective unimolecular aggregates in water. Furthermore, we have measured TEM for the 10:1 mixture. The obtained image (Supplementary Fig. 15) differs considerably from that observed for the 2:1 mixture, but resembles that obtained from pure PBI **1** in water (Fig. 2a). Thus, we can conclude that minor amounts of PBI **2** cannot transform the morphology from stiff segmented nanorods (as observed for pure PBI **1**) into flexible elongated nanowires (as observed for 2:1 mixed aggregates). On the other hand, the use of >50% of PBI **2** in the mixture results in colloidal samples, whose suspensious character is clearly visible by the eyes. Clearly, such large amounts of PBI **2** cannot be embedded homogeneously in PBI **1** aggregates and accordingly similar aggregates like those of pure PBI **2** are formed. From these studies we can deduce that homoaggregated blocks of PBI **1** and PBI **2**, respectively, are given for all accessible mixing ratios according to ultraviolet–visible spectroscopy, but that morphologically distinct macroscopic nanowires as shown in Fig. 4 are only possible in a narrow mixing regime around 2:1 molar ratio.

Further insight into the most interesting 2:1 composition of the mixed PBI **1**/PBI **2** nanowire system is obtained by fluorescence spectroscopy. The nanowires formed by co-assembly of PBI **1** and PBI **2** display an intense fluorescence (Fig. 5, Supplementary Figs 16 and 17) with fluorescence quantum yield (Φ) of 6.4%, which is higher than the value for both PBI **1** nanorods (Φ=1.7%; ref. 27) and PBI **2** nanoworms in water (Φ=2.2%). Excitation-dependent fluorescence spectra (Supplementary Fig. 18) show that the fluorescence spectrum obtained upon excitation at the shortest wavelength absorption maximum (480 nm) affords the bathochromically displaced excimer emission at 650 nm that undoubtedly originates from PBI **1** aggregates, whereas the fluorescence spectrum obtained upon excitation at the red-shifted maximum (600 nm) shows a rather similar emission as observed for pure PBI **2** nanoworms (Fig. 3). Accordingly, as for the absorption spectra, also the emission spectra corroborate the idea that the nanowires are composed of alternating segments of PBI **1** and PBI **2** aggregates, that is, self-sorting on the nanoscopic scale is involved in the co-assembly process due to the very distinctive structural features of PBI **1** (flat aromatic core) and PBI **2** (twisted aromatic core)^47^,^48^. Furthermore, the emission spectra indicate a lack of energy transfer between the respective blocks. At a first glance this is a very surprising result for heteroaggregates composed of short blocks, that is, close neighbourhood of PBI **1** and PBI **2** segments. However, we have to consider the following unusual situation in these heteroaggregates: PBI **1** aggregate has the higher-lying S_1_ state than PBI **2** aggregate, but the emission of PBI **1** aggregates takes place from a lower-lying excimer state^44^, as evidenced by a characteristic broad emission band at longer wavelength, that is, lower energy. Owing to the ultrafast relaxation into this excimer state^44^, photoexcited PBI **1** segments will accordingly not transfer their energy to neighbouring segments of PBI **2**. On the other hand, if the latter are photoexcited, they can also not transfer their energy to PBI **1** segments because there is no overlap of the emission band of PBI **2** segments with the absorption band of PBI **1** segments due to the more hypsochromic shift of the latter.

### Self-sorting of PBI 1 and PBI 2

**Figure 6:**
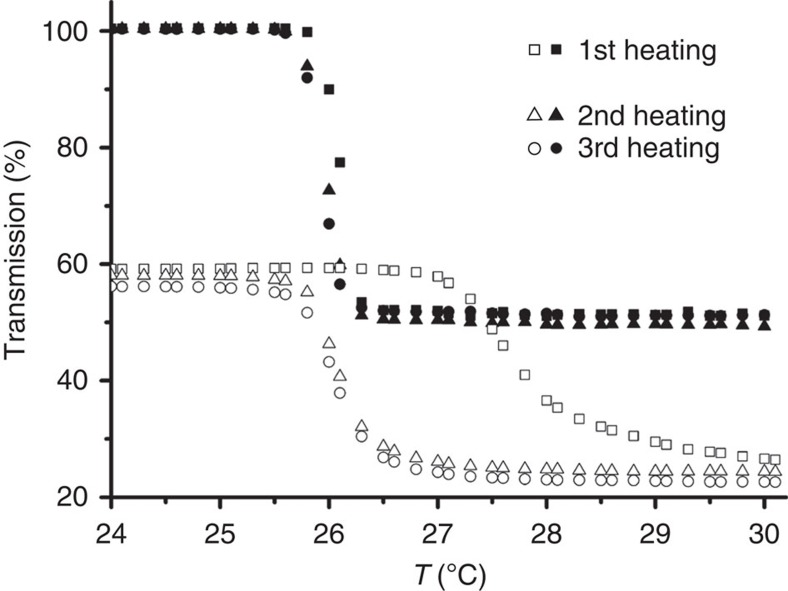
LCST behaviour of co-assembled PBI 1/PBI 2 nanowires and unimolecular PBI 1 self-assemblies in water. Determination of LCSTs for nanowires (open symbols) formed by co-assembly of PBI **1** and PBI **2** in a molar ratio of 2:1 ([PBI **1**]=5 × 10^−5^ M in water) and for unimolecular PBI **1** aggregates (closed symbols, [PBI **1**]=5 × 10^−5^ M). Transmission was monitored at 800 nm. Heating rate was 0.1 °C min^−1^.

Our studies so far indicate that the mixed PBI **1**/PBI **2** system is composed of alternating small-sized segments of homoaggregated PBI **1** and homoaggregated PBI **2** that form ultralong nanowires composed of thousands of stacked molecules. If we assume the presence of the smallest possible segment size for PBI **2**, that is, dimer, we can calculate a block size of four PBI **1** homoaggregates at the chosen 2:1 molecular ratio (for model see Fig. 4c). The remaining question is now whether these nanowires constitute a thermodynamically stable state or they were formed under kinetic control upon evaporation of THF from the THF/H_2_O solvent mixture. To address this question we subjected the 2:1 mixed nanowire system to heating–cooling cycles (Fig. 6). Upon first heating, a phase transition for the mixed system occurred at about 27.5 °C, whereas further cooling–heating cycles showed a phase transition at a lower temperature of 26 °C, the latter being almost identical to that of pure PBI **1** aggregates in water. These phase transitions originate from a pronounced conformational change of the oligoethylene glycol (OEG) chains and concomitant partial desolvation^49^. This phenomenon leads to the unexpected precipitation upon heating of OEG-functionalized hydrophobic molecules and is characterized by the respective phase-transition temperature that is called the lower critical solution temperature (LCST). Due to the heating above its LCST of 27.5 °C, PBI **1**/PBI **2** mixed nanowires are not preserved during the first phase transition. Instead, PBI **2** sediments upon re-cooling, whereas PBI **1** is re-dissolved as confirmed by subsequent ultraviolet–visible measurements that show merely the absorbance of PBI **1** aggregates. A similar separation of PBI **1** and PBI **2** was observed when a different preparation method was applied, namely the addition of water to a perfectly mixed solid material consisting of a 2:1 mixture of PBI **1** and PBI **2**. In this case only PBI **1** is dissolved. Thus, these results unambiguously prove that the mixed PBI **1**/PBI **2** nanowire system obtained upon evaporation of THF from a THF/H_2_O solvent mixture constitutes a kinetically trapped self-assembly product. Notably, pure PBI **2** in water is not subject to LCST behaviour because it does not dissolve in pure water as discussed above. Thus, after the first heating cycle, the co-assembled system is transformed into dissolved PBI **1** aggregates and dehydrated suspension-forming PBI **2** aggregates. Because the transmission of the mixed system at 800 nm can be completely regenerated during the heating cycles, this change in transmission can only be attributed to the PBI **1** homoaggregate system, which undergoes phase transition.

The integration of PBI **2** dimers in 1D PBI **1** aggregates during the co-self-assembly process prohibits a pronounced elongation of the PBI **1** homostacks into stiff segmented nanorods. Statistically, four PBI **1** molecules interact with each other at the 2:1 ratio of PBI **1** with dimers of PBI **2**, which might be the reason for the higher LCST of the mixed system compared with that of PBI **1** homoaggregates. The presence of PBI **2** further prevents the fusion of 1D PBI **1** nanorods into lamellar structures as it is the case for pure PBI **1** (ref. 31). This kinetically stabilized state can be transformed into the thermodynamic one in water by heating, leading to self-sorted and well-dissolved PBI **1** homoaggregates and PBI **2** precipitate.

Since LCST is an entropic phenomenon, the higher LCST of the mixed PBI **1**/PBI **2** system implies that the nanowires created from **1** and **2** are of different order compared with the homoaggregates of PBI **1**. These differences may be attributed to the mutual interaction^50^ of planar and core-twisted PBI molecules in the nanowires. The slow evaporation of THF during nanowire preparation enforces both PBIs to mix with each other. This is presumably supported by the similarity in molecular structure, especially with regard to the OEG-bearing imide substituents, that guarantee solubility at the given experimental conditions. The trend of the binding constants (Supplementary Fig. 6) suggests that with increasing water content PBI **2** reaches a fully aggregated but only dimeric state, while PBI **1** aggregation into elongated columnar structures is by far not complete. This state is supposed to be very close to the end point of nanofiber formation. As mentioned before, PBI **2** dimers obviate further interactions with each other via π–π stacking because of steric hindrance of the bulky aryloxy bay substituents. To overcome the unfavourable exposure of π-surfaces of such dimers to the aqueous environment, co-assembly with sterically less encumbered PBI **1** takes place as evidenced by 2D NMR spectroscopy (Fig. 4). Thus, 1D block copolymer structures are formed, where PBI **2** dimers and PBI **1** assemblies of variable size (depending on the excess of PBI **1**) are alternately distributed.

Because PBI **2** molecules are not soluble in pure water at any temperature, it is reasonable to assume that PBI **1** replaced THF as a solubilizing environment upon evaporation of THF from THF/H_2_O mixture during the co-assembly process. Finally, at higher water, content kinetic trapping occurs mainly because of the much stronger hydrophobic interactions constraining the reversibility^46^. Because of its pronounced hydrophobicity, PBI **2** looses its solvation shell along the way from monomeric state to being part of the nanowires. Thus, the mixed structure remains stable even in pure water due to strong cohesive forces between planar and core-twisted PBIs, although PBI **2** alone cannot exist in the given solvent and prevails in a non-hydrated ‘precipitated' state.

## Discussion

This study has elucidated the co-self-assembly of two amphiphilic PBI dyes that bear identical OEG side chains for solubilization in water but are distinguished by the π-conjugated core that is planar for PBI **1** and twisted (as well as more hydrophobic) for PBI **2**. Owing to the different structural features and hydrophobicity, PBI **1** exhibits remarkable solubility at room temperature in water even at quite high concentrations, whereas the core-twisted PBI **2** can only form suspension-forming self-assemblies of small size in water. In the presence of PBI **1**, however, co-assembly with **2** becomes possible upon evaporation of the co-solvent THF, providing ultrathin and micrometre-long nanowires with uniform diameter of 3–4 nm. Our studies showed that these co-assemblies consist of an alternating sequence of respective self-assembled blocks of PBI **1** and PBI **2** that exhibit unusual fluorescence properties, that is, depending on the excitation wavelength emission originates from either PBI **1** or PBI **2** segments, respectively. Such mixed dye assemblies can only be formed by means of a kinetically controlled pathway during passing through the THF/water gradient upon THF evaporation. After formation, these co-assemblies are persistent in pure water due to strong hydrophobic interactions among PBI **1** and PBI **2** molecules that are co-assembled in ultrathin nanowires. However, desolvation of PBI **1** upon heating (LCST behaviour) destabilizes the nanowires, thus ending in the thermodynamically favoured self-sorting of both PBI components into their respective homoaggregates. Our studies demonstrate that mixed supramolecular block copolymers can be generated by applying appropriate sample preparation conditions, that is, strong cohesive forces in water. On the basis of our results we can conclude that co-assembly of two-component systems can follow different pathways than that of their individual components, facilitating the creation of nanosystems with different morphology and properties. This principle may be applied to other multicomponent self-assembled nanosystems whose functions may ultimately bridge the gap to the amazing examples found in nature.

## Methods

### Preparation of PBI aggregates

PBI **2** aggregates: deionized water (1.0 ml) was added dropwise into a THF solution of PBI **2** (50 μl, 1.0 mg ml^−1^) to induce the aggregate formation. The aggregate suspension was exposed to air to remove THF at room temperature for several days until it is condensed to 0.65 ml, leading to a final concentration of 0.077 mg ml^−1^ for PBI **2** in deionized water.

PBI **1**/PBI **2** co-aggregates: deionized water (0.5 ml) was added dropwise into the mixed THF solution of PBI **1** (25 μl, 1.0 mg ml^−1^) and PBI **2** (17 μl, 1.0 mg ml^−1^); [PBI **1**]:[PBI **2**]=2:1 in molar ratio. The aggregate suspension was exposed to air to remove THF at room temperature for several days until it is condensed to 0.05 ml, leading to a final concentration of 0.5 mg ml^−1^ for PBI **1** in deionized water.

For further information on Methods, see the Supplementary Methods.

## Author contributions

F.W. initiated and guided the research programme; D.G. synthesized the molecules; D.G. and X.Z. performed the spectroscopy and TEM investigations; V.S. performed the atomic force microscopy and cryo-TEM experiments; X.Z. designed the graphical artwork; D.G. and F.W. wrote the manuscript.

## Additional information

**How to cite this article:** Görl, D. *et al*. Supramolecular block copolymers by kinetically controlled co-self-assembly of planar and core-twisted perylene bisimides. *Nat. Commun.* 6:7009 doi: 10.1038/ncomms8009 (2015).

## Supplementary Material

Supplementary Information Supplementary Figures 1-18, Supplementary Methods and Supplementary
