# Supplementary material for: Supramolecular block copolymers by kinetically controlled co-self-assembly of planar and core-twisted perylene bisimides
Source: Nat Commun. 2015 May 11;6:7009. doi: 10.1038/ncomms8009 (PMC4432616; doi:10.1038/ncomms8009)
Supplement: Supplementary Information — Supplementary Figures 1-18, Supplementary Methods and Supplementary [file ncomms8009-s1.pdf]

SUPPLEMENTARY FIGURES

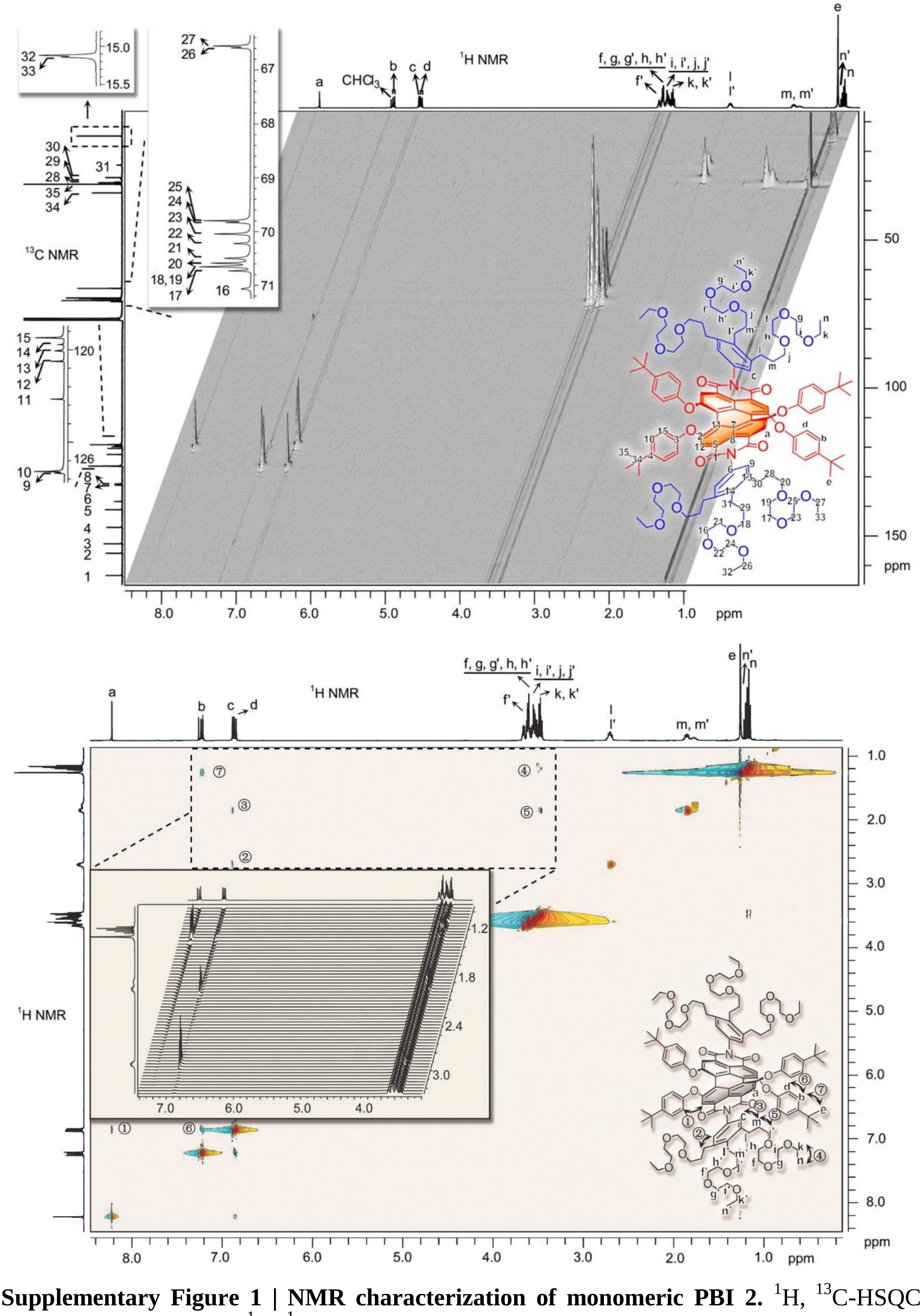

Supplementary Figure 1 | NMR characterization of monomeric PBI 2. ^{1}H, ^{13}C-HSQC NMR spectra (top) and ^{1}H, ^{1}H-ROESY NMR spectra (bottom) of core-twisted PBI **2** with corresponding signal assignments. ^{1}H and ^{13}C NMR spectra are also shown in ^{1}H, ^{13}C-HSQC NMR spectra.

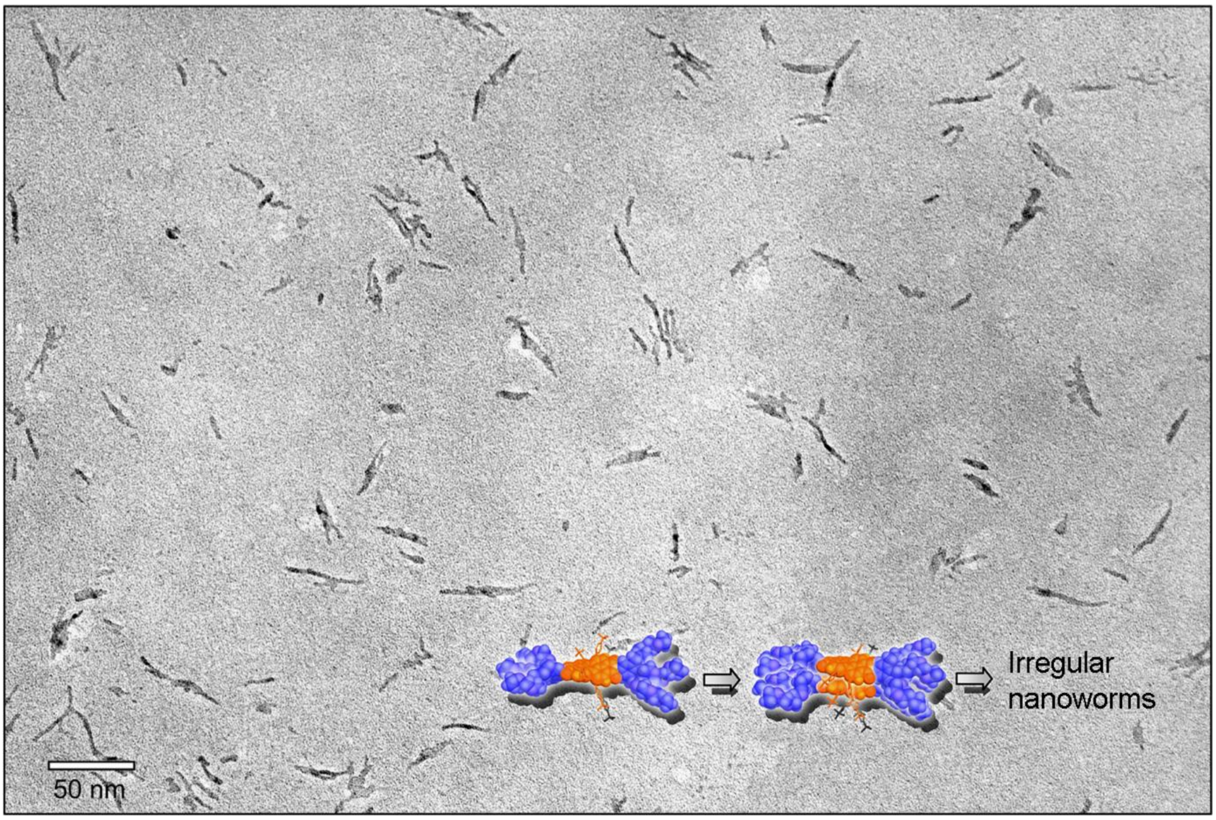

Supplementary Figure 2 | Morphology of PBI 2 self-assemblies. TEM image of PBI **2** aggregates prepared in water. [PBI **2**] = 3 x 10^{-5} M (0.077 mg/mL). Inset: Schematic illustrations of PBI **2** assemblies based on space-filling (CPK) model.

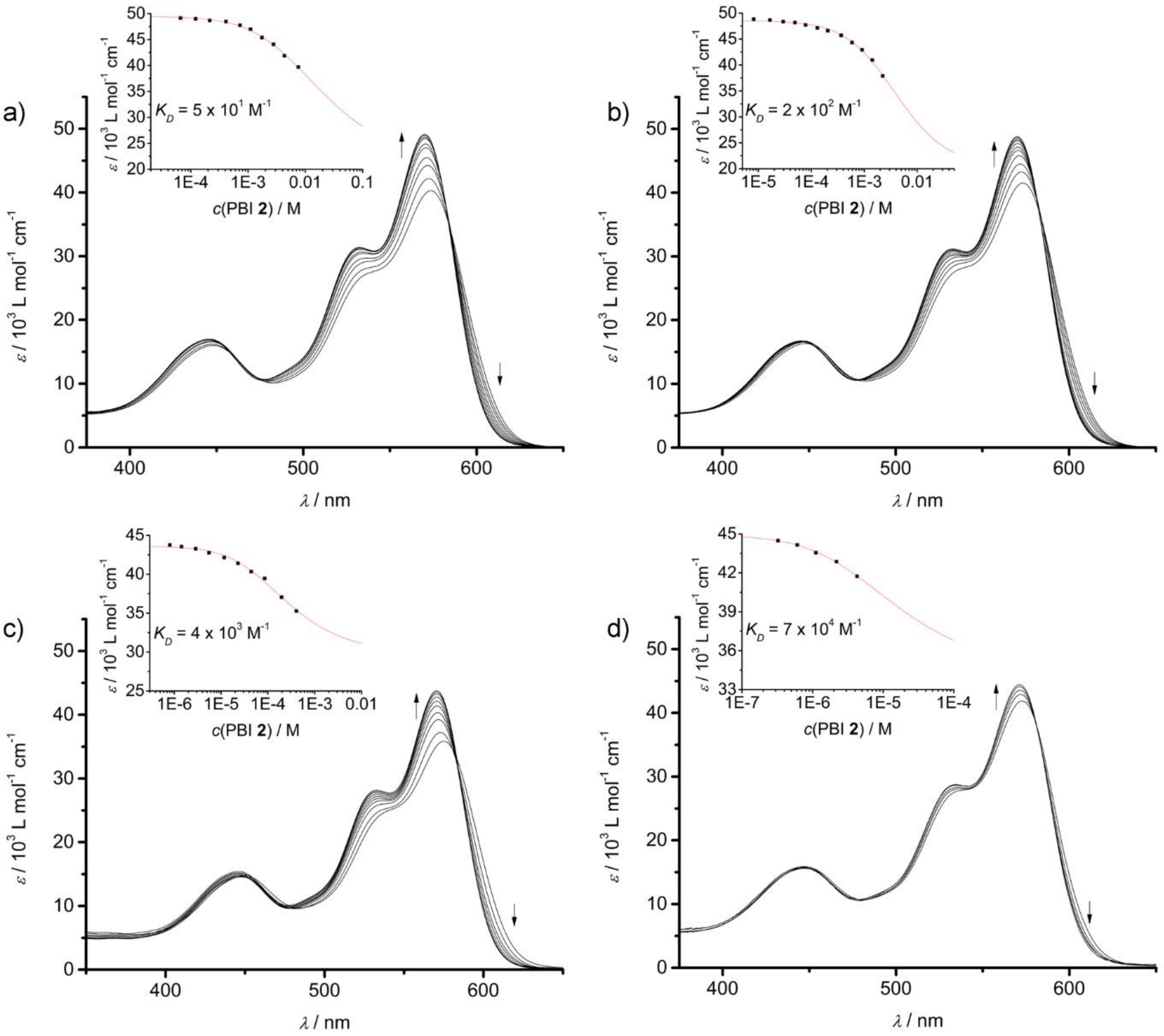

Supplementary Figure 3 | Self-assembly of PBI 2 in THF/H_2O mixtures. Concentration-dependend UV-vis absorption spectra of PBI **2** in THF/H_2O mixtures with 70% (a), 60% (b), 50% (c) and 40% (d) THF content at 22 °C ([PBI **2**] = 7.52 x 10^{-3} – 6.62 x 10^{-5} M (a), 1.42 x 10^{-3} – 8.17 x 10^{-6} M (b), 4.05 x 10^{-4} – 8.03 x 10^{-7} M (c), 4.27 x 10^{-6} – 3.25 x 10^{-7} M (d)). Arrows indicate spectral changes upon concentration decrease. Insets: Plots of extinction of the monomeric absorbance maximum (at 570 nm (a, b), 571 nm (c, d)) vs. concentration of PBI **2** and fitting curve of the data points according to the dimerization model.[1]

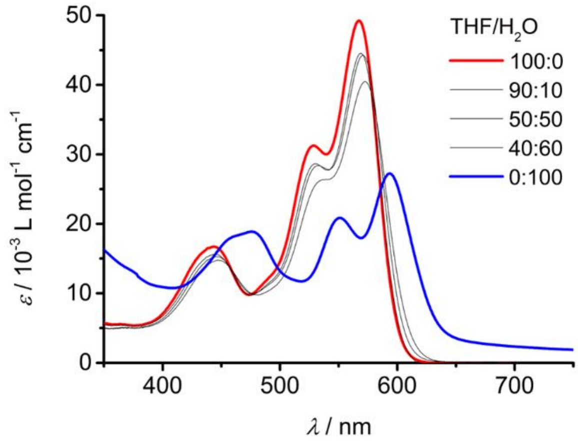

Supplementary Figure 4 | Solvent-dependent UV-vis spectra of PBI 2. UV-vis absorption spectra of 2.0 x 10^{-6} M solutions of PBI **2** in THF/water mixtures at 20 °C. Concentration in pure H_2O was 3 x 10^{-5} M.

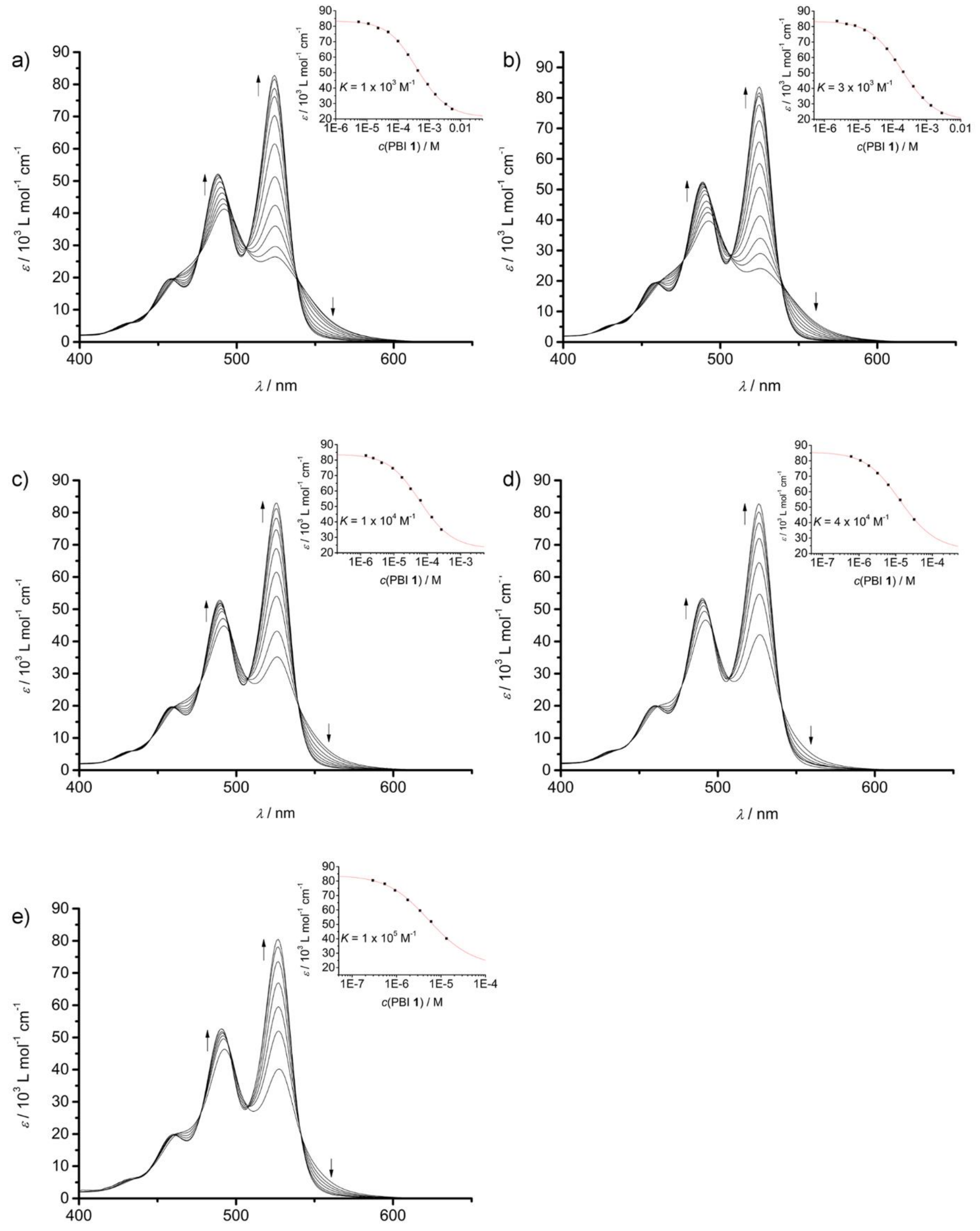

Supplementary Figure 5 | Self-assembly of PBI 1 in THF/H_2O mixtures. Concentration-dependent UV-vis spectra of PBI **1** in THF/H_2O mixtures with 70% (a), 60% (b), 50% (c), 40% (d) and 35% (e) THF content at 22 °C ([PBI **1**] = 5.35 x 10^{-3} – 5.45 x 10^{-6} M (a), 2.77 x 10^{-3} – 2.34 x 10^{-6} M (b), 2.66 x 10^{-4} – 1.45 x 10^{-6} M (c), 3.19 x 10^{-5} – 6.12 x 10^{-7} M (d), 1.33 x 10^{-5} – 2.92 x 10^{-7} M (e)). Arrows indicate spectral changes upon concentration decrease. Insets: Plots of extinction of the monomeric absorbance maximum (at 524 nm (a), 525 nm (b), 526 nm (c, d), 527 nm (e)) vs. concentration of PBI **2** and fitting curve of the data points according to the isodesmic model.[1]

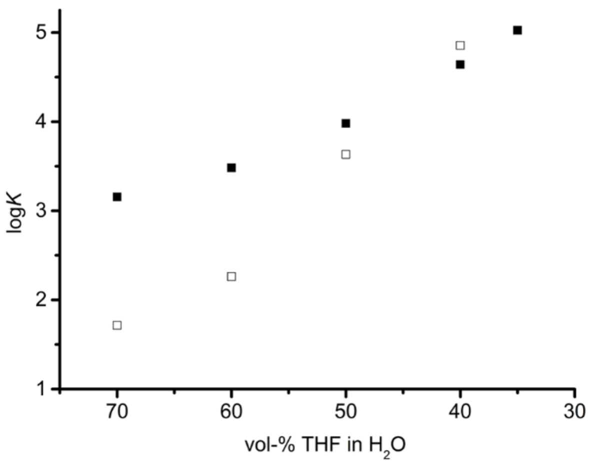

Supplementary Figure 6 | Self-assembly of PBI 1 and PBI 2 in THF/H_2O mixtures. Plot of logarithmic binding constants for PBI **1** (closed symbols) and PBI **2** (open symbols) versus THF content in water. Binding constants have been obtained from concentration-dependent UV-vis spectra at 22 °C and calculated according to the isodesmic (PBI **1**) and dimerization (PBI **2**) model.

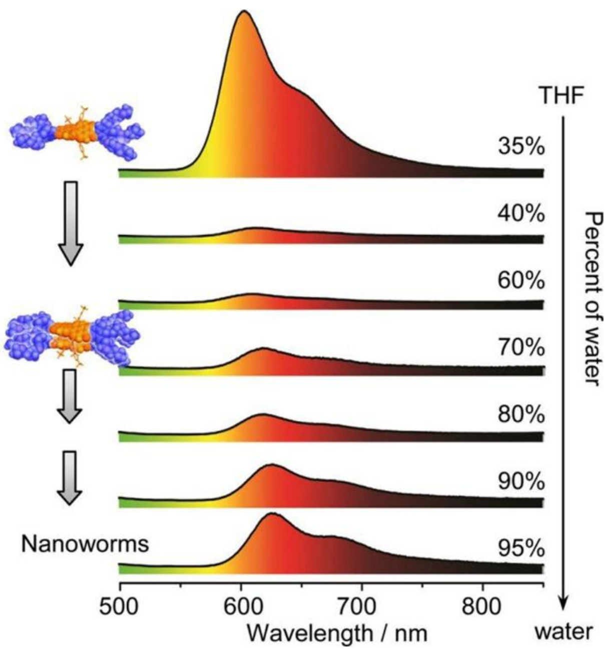

Supplementary Figure 7 | Solvent-dependent fluorescence spectra of PBI 2. Fluorescence spectra of PBI **2** assemblies in THF/water mixtures with increasing water content leading to nanoworm formation; [PBI **2**] = 2 x 10^{-6} M; λ_{ex} = 455 nm.

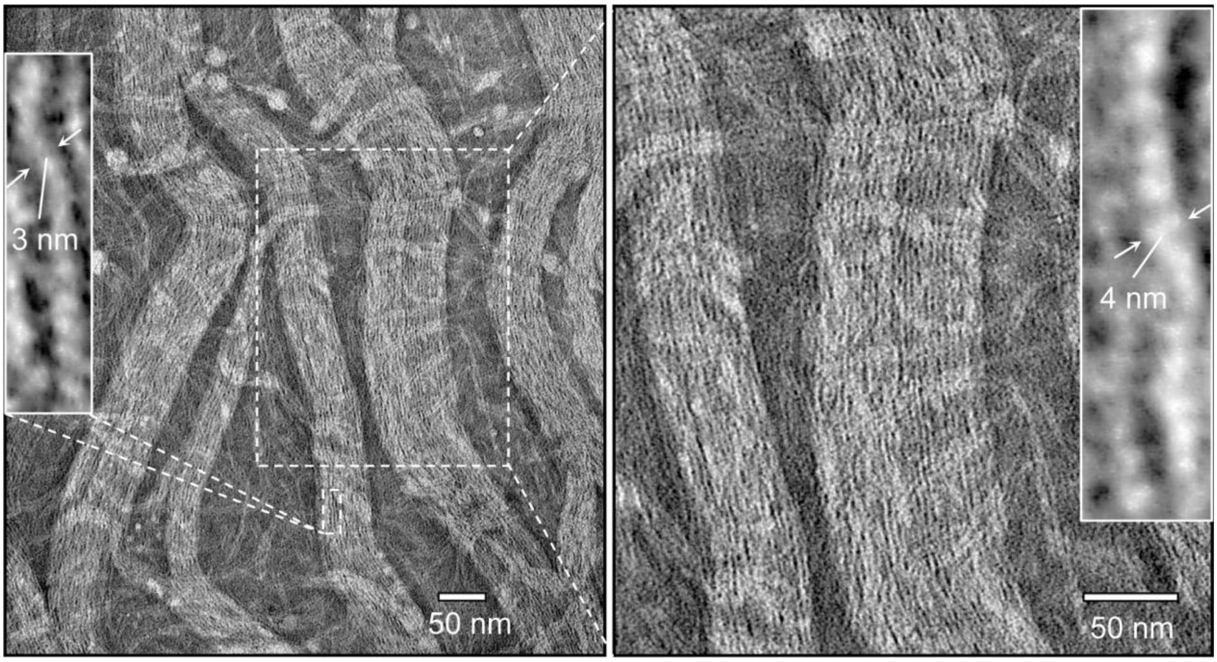

Supplementary Figure 8 | Morphology of PBI 1/PBI 2 co-assemblies. TEM images of nanowires prepared from co-assembly of PBI **1**/PBI **2** in a molar ratio of 2:1; [PBI **1**] = 3.2 x 10^{-4} M (0.5 mg/mL). Inset: Magnified TEM image of the single-molecule-thin segmented nanowires.

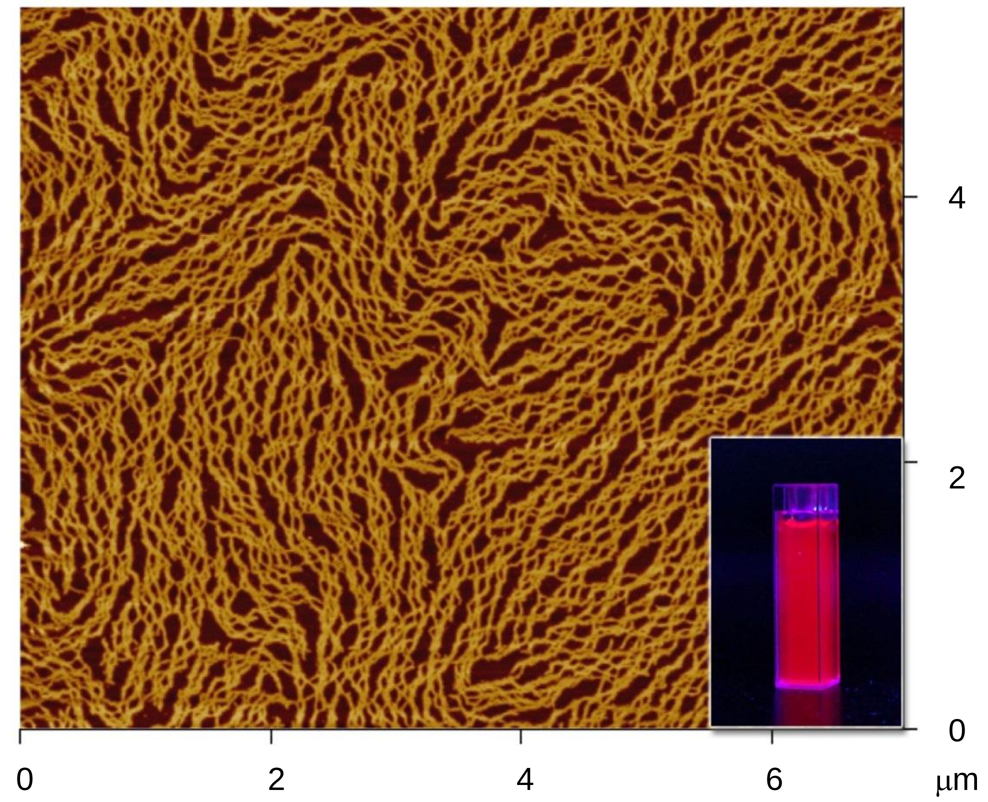

Supplementary Figure 9 | Morphology of PBI 1/PBI 2 co-assemblies. AFM height image of PBI **1**/PBI **2** co-aggregates on mica prepared from water solution; [PBI **1**] : [PBI **2**] = 2 : 1 in molar ratio, [PBI **1**] = 3.2 x 10^{-4} M. Inset: Photograph of PBI **1**/PBI **2** co-aggregates in water under ultraviolet light.

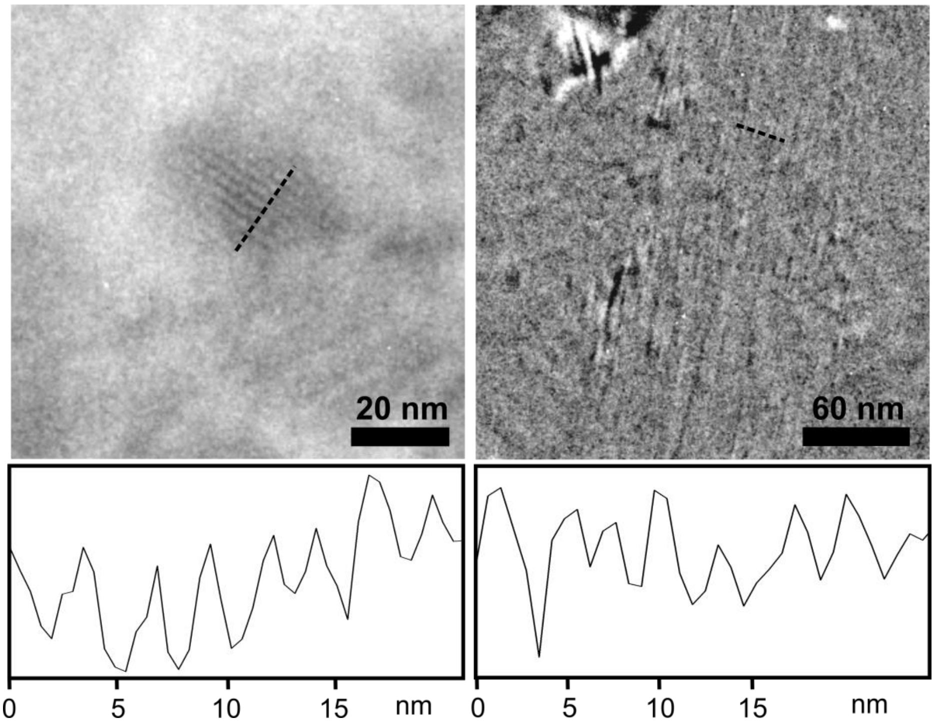

Supplementary Figure 10 | Morphology of PBI 1/PBI 2 co-assemblies. Cryo-TEM images (top) of nanowires prepared from co-assembly of PBI **1**/PBI **2** in a molar ratio of 2:1; [PBI] = 7.5 x 10^{-5} M. Bottom: Corresponding cross-section analysis along the black dashed lines.

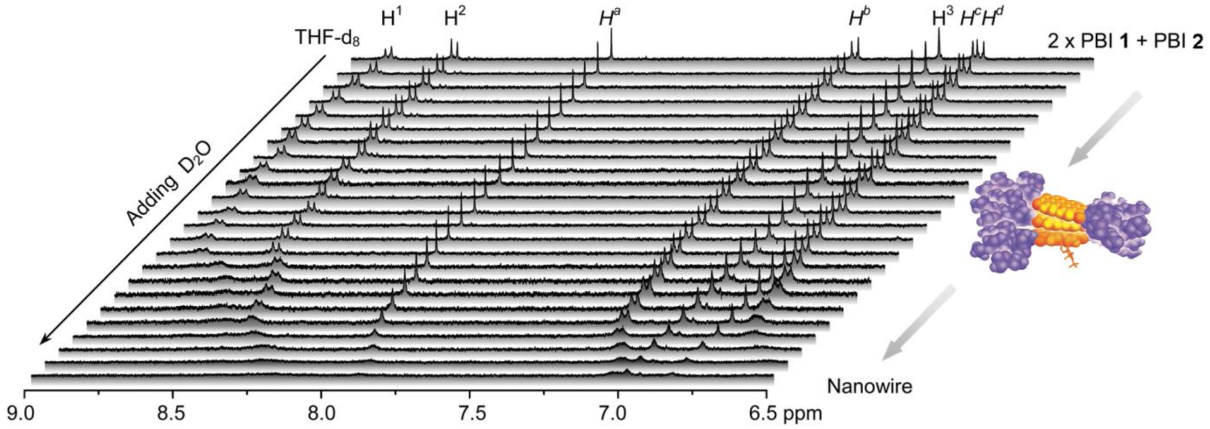

Supplementary Figure 11 | NMR spectra for PBI 1/PBI 2 co-assembly process. ^{1}H NMR spectra of PBI **1**/PBI **2** co-assembly in D_2O/[D_8]THF with D_2O content from 0 % to 66 % by adding portions of 10 μL D_2O; [PBI **1**] = 2.6 x 10^{-4} M, [PBI **1**] : [PBI **2**] = 2 : 1 in molar ratio.

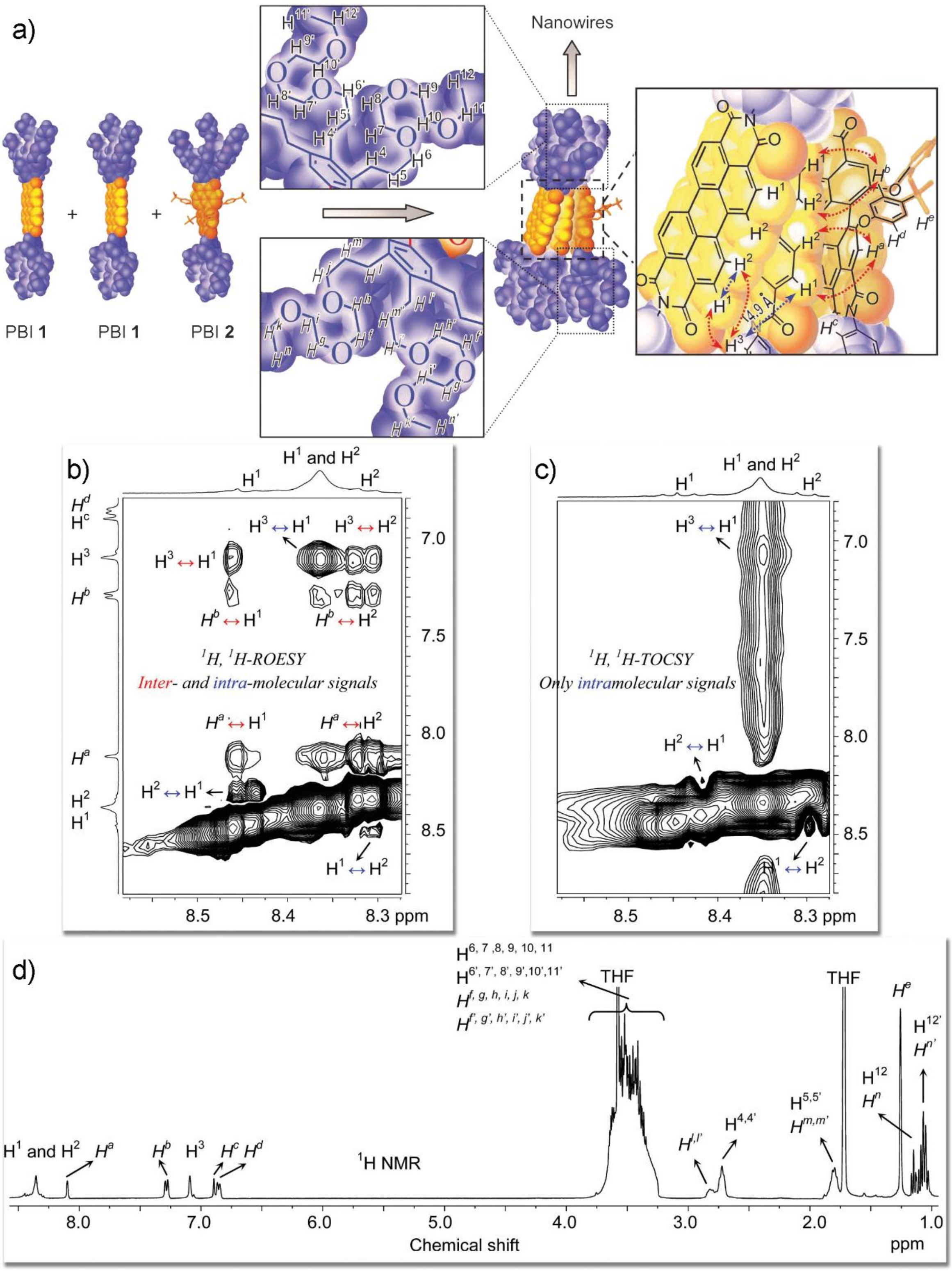

Supplementary Figure 12 | Characterization of PBI 1/PBI 2 co-assemblies by two-dimensional NMR spectroscopy. a) CPK model from AM1 calculation for PBI **1**/PBI **2** co-assembling intermediate of nanowires. ^{1}H, ^{1}H-ROESY (b), ^{1}H, ^{1}H-TOCSY (c), and ^{1}H NMR (d) spectra of PBI **1**/PBI **2** co-assemblies in D_2O/THF-d_8 (400 μL/ 600 μL). [PBI **1**]= 2.6 x 10^{-4} M, [PBI **1**] : [PBI **2**] = 2 : 1 in molar ratio.

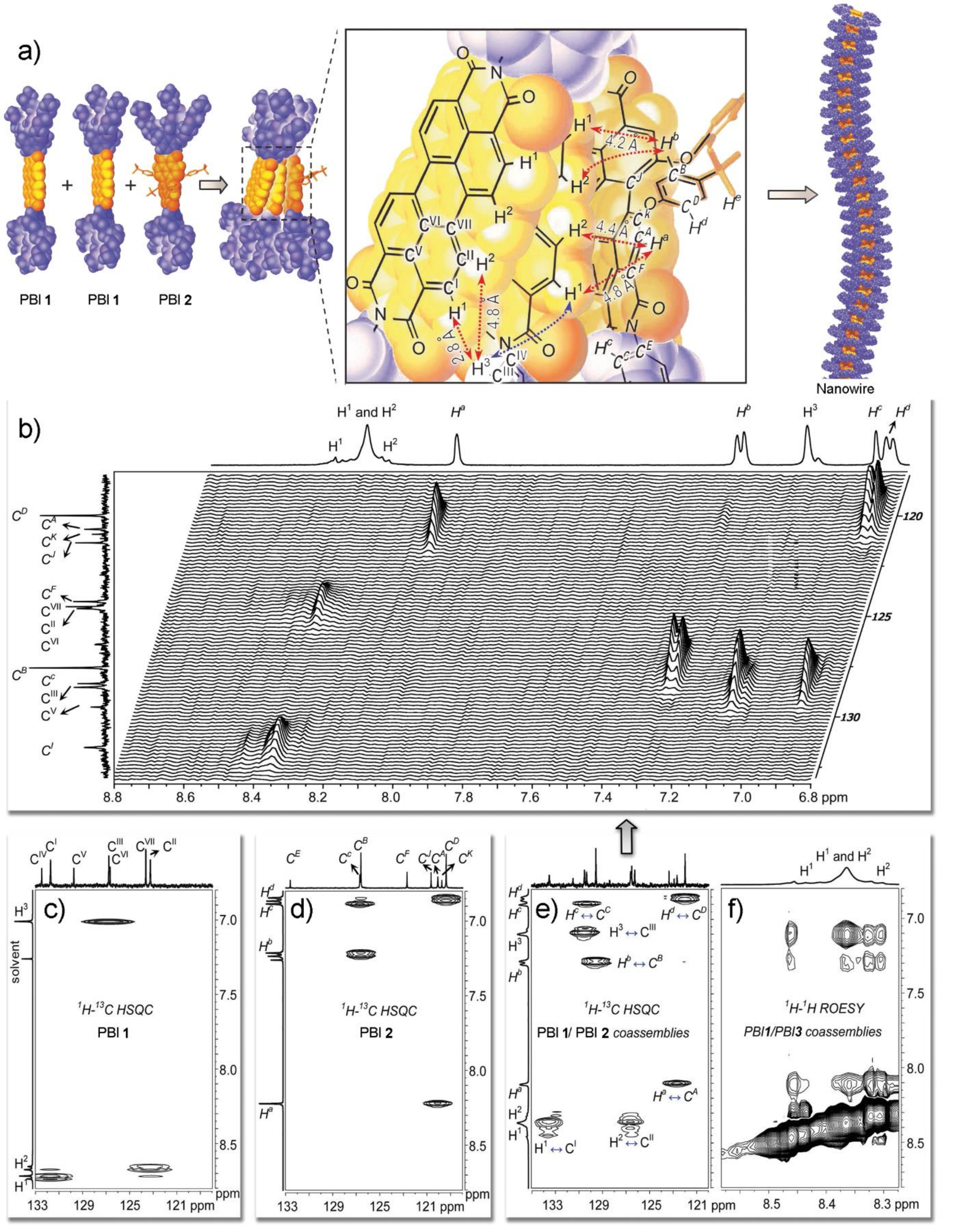

Supplementary Figure 13 | Characterization of PBI 1/PBI 2 co-assemblies by two-dimensional NMR spectroscopy. a) CPK model from AM1 calculation for the PBI **1**/PBI **2** co-assembling intermediate of nanowires. b) and e) ^{1}H, ^{13}C-HSQC spectrum of one PBI **1**/PBI **2** co-assembling segment of nanowires in D_2O/[D_8]THF (400 μL/ 600 μL), [PBI **1**]= 4.9 x 10^{-3} M, [PBI **1**] : [PBI **2**] = 2 : 1 in molar ratio. c) ^{1}H, ^{13}C-HSQC spectrum of only PBI **1** in $CDCl_3$. d) ^{1}H, ^{13}C-HSQC spectrum of only PBI **2** in $CDCl_3$. e, f) ^{1}H, ^{13}C-HSQC and ^{1}H, ^{1}H-ROESY spectrum of the PBI **1**/PBI **2** co-assembling intermediate of nanowires in the D_2O/[D_8]THF (400 μL/ 600 μL); [PBI **1**] = 4.9 x 10^{-3} M, [PBI **1**] : [PBI **2**] = 2 : 1 in molar ratio.

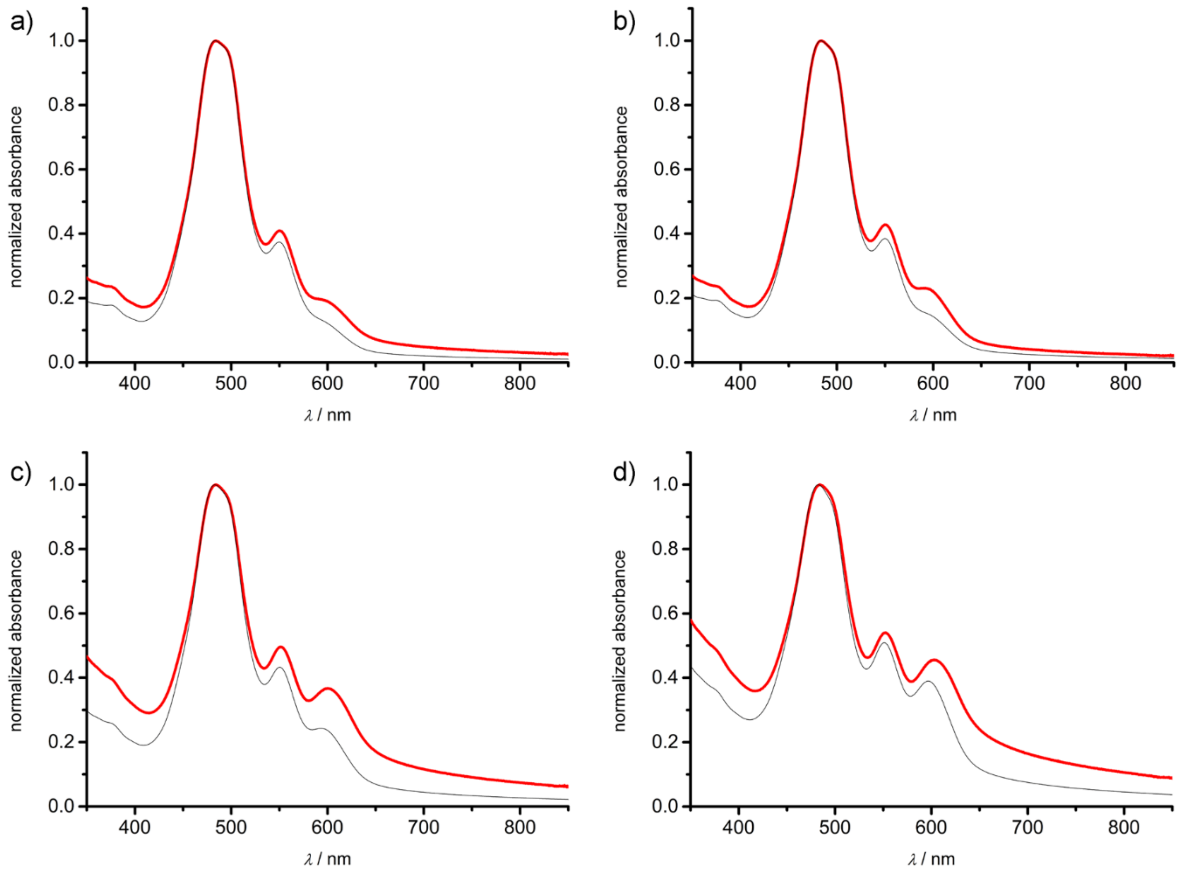

Supplementary Fig. 14 | Optical properties of PBI 1/PBI 2 mixtures. Normalized absorption spectra (red) of co-assemblies of PBI **1**/PBI **2** in a molar ratio of 10:1 (a), 8:1 (b), 4:1 (c) and 2:1 (d) in water and calculated absorption spectra (black) for the respective mixtures based on the linear superposition of normalized absorption spectra of PBI **1** and PBI **2** in water.

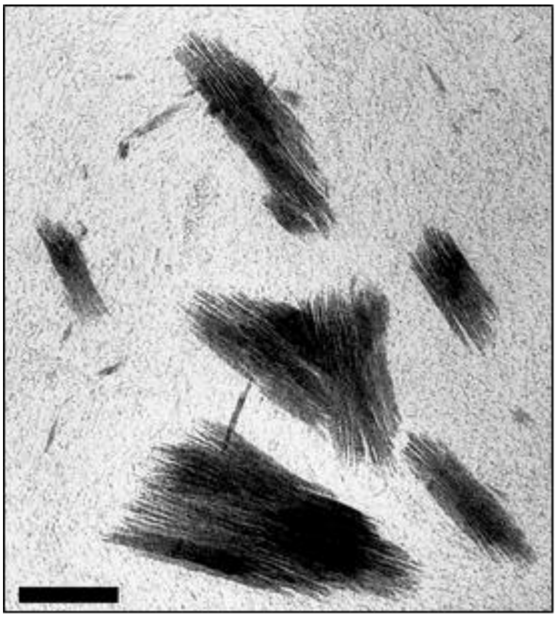

Supplementary Fig. 15 | PBI 1 and PBI 2 co-assemblies in water. TEM image of co-assemblies of PBI **1** and PBI **2.** [PBI **1**]:[PBI **2**] = 10:1 in molar ratio, [PBI **1**] = 0.5 mg /mL. Scale bar is 100 nm.

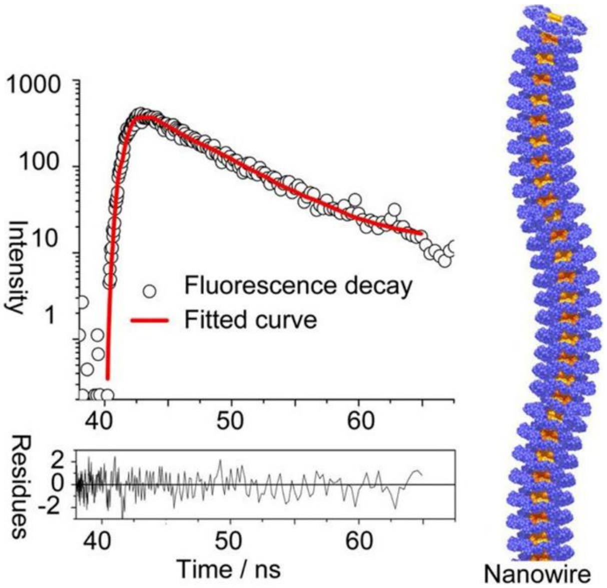

Supplementary Figure 16 | Time-resolved fluorescence spectrum of PBI 1/PBI 2 co-assemblies. Fluorescence decay of nanowires formed from PBI **1**/PBI **2** (2:1 molar ratio; [PBI **1**] = 0.5 mg/mL) co-assembly in water. λ_{ex} = 579 nm, λ_{em} = 630 nm, fluorescent lifetime $<\tau>$ = 0.6 ns (τ1) x 48.3 % + 4.7 ns (τ2) x 51.7 % = 2.7 ns; χ^2 = 1.1.

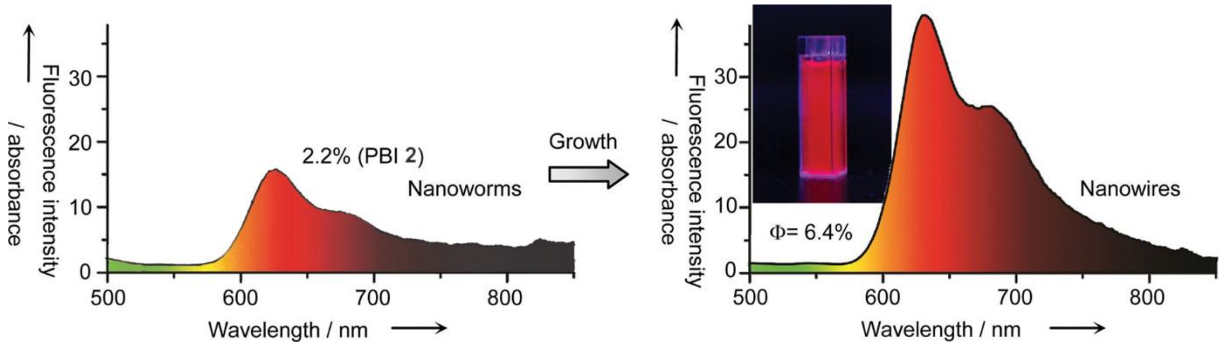

Supplementary Figure 17 | Photoluminescence properties of perylene dye aggregates in water. Left: Fluorescence spectrum of nanoworms in water, [PBI **2**] = 0.077 mg/mL. Right: Fluorescence spectrum of nanowires in water, PBI **1** : PBI **2** = 2 : 1 in molar ratio, [PBI **1**] = 0.5 mg/mL. Inset: Photograph of nanowires in water under ultraviolet light. Fluorescence quantum yields (Φ) are indicated in figures.

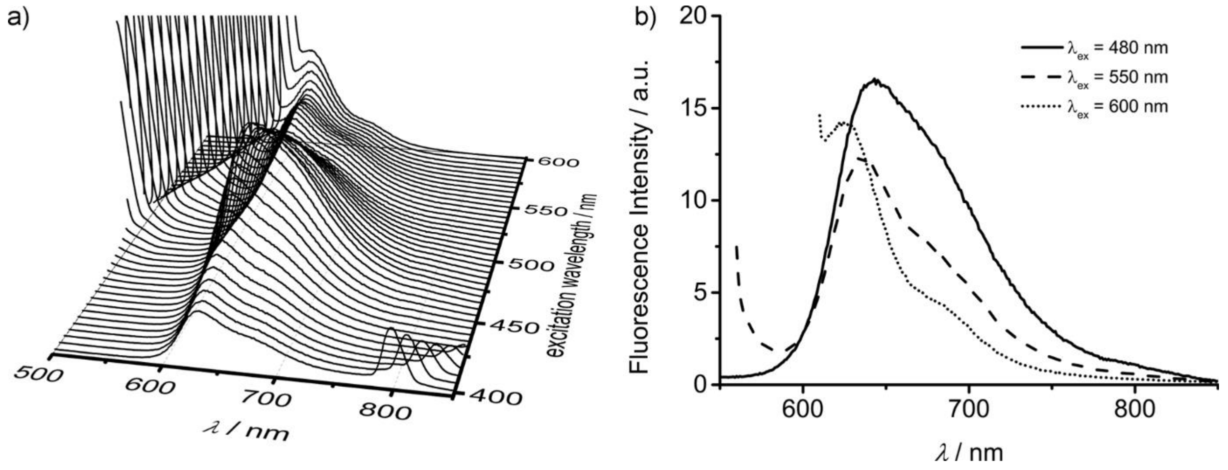

Supplementary Figure 18 | Fluorescence spectra of PBI 1/PBI 2 co-assemblies. a) Excitation-dependent 3D fluorescence spectra of nanowires formed by co-assembly of PBI **1**/PBI **2** in a molar ratio of 2:1 ([PBI] = 7.5 x 10^{5} M in water). Magic-angle setup was used. b) Comparison of the corresponding fluorescence spectra excited at 480 nm, 550 nm and 600 nm.

SUPPLEMENTARY METHODS

1. Materials

1,6,7,12-Tetra(4-*tert*-butylphenoxy)perylene-3,4:9,10- tetracarboxylic acid bisanhydride used as precursor of PBI **2** was synthesized according to literature.[2] Perylene bisimide (PBI) **1** was synthesized according to our previously reported procedure.[3] PBI **2** is a new compound, and its synthesis is described in next Section.

2. Methods

NMR spectroscopy. ^{1}H NMR, ^{13}C NMR, ^{1}H, ^{13}C-Heteronuclear Single Quantum Correlation (HSQC), ^{1}H, ^{13}C-Heteronuclear Multiple Bond Correlation (HMBC), and ^{1}H-^{1}H ROESY (Rotating-frame Nuclear Overhauser Effect Spectroscopy) were recorded at 300 K on a Bruker Advance 400 (400 MHz) spectrometer using $CDCl_3$ or D_2O as solvent. ^{1}H NMR and ^{13}C NMR signals were assigned with the aid of two-dimensional ^{1}H, ^{13}C-HSQC and ^{1}H, ^{13}C-HMBC spectra.[4] Multiplicities for proton signals are abbreviated as s, t, and m for singlet, triplet, and multiplet, respectively.

UV-vis spectroscopy. UV-vis absorption spectra were recorded in spectroscopic grade organic solvents (Uvasol®) dichloromethane and tetrahydrofuran as well as in millipore water (Merck) by using a Perkin Elmer Lambda 40P spectrophotometer. The spectra were measured in quartz glass cuvettes under ambient conditions and the extinction coefficients ε were calculated from Lambert-Beer's law. LCSTs in water were determined by recording the transmission at 800 nm in 10 mm quartz cuvettes using heating rate of 0.1 °C/min.

Fluorescence spectroscopy. The steady-state fluorescence spectra in solution were recorded under ambient conditions on a PTI QM4-2003 fluorescence spectrometer and corrected against photomultiplier and lamp intensity. The slit width of both monochromators

was between 5.0 and 8.0 nm. Horizontally set up polarizers were used in the case of monomeric samples, and magic-angle setup in case of aggregated species.[5, 6] Fluorescence quantum yields (Φ) of monomeric PBIs **2** and **3** were calculated from the integrated intensity under the emission band (I) using the following equation:

$$\Phi = \Phi_r \frac{I}{I_r} \frac{OD_r}{OD} \frac{n^2}{n_r^2} \quad \mathbf{(1)}$$

where *OD* is the optical density of the solution at the excitation wavelength and *n* is the refractive index. The optical density of the solution for the calculation of quantum yields was less than 0.1 at the excitation wavelength. *N*,*N*'-Bis(2,6-diisopropylphenyl)-1,6,7,12-tetraphenoxyperylene-3,4:9,10 -tetracarboxylic acid bisimide (Φ_r = 0.96) in chloroform was used as reference.[7]

Fluorescence lifetimes were measured on a PTI Laser Strobe fluorescence lifetime spectrometer equipped with a PTI GL-3300 nitrogen laser using a dye laser PTI GL302 as an excitation source and stroboscopic detection. The instrument response function was collected by scattering the exciting light of aqueous colloid silica (LUDOX). Fluorescence decay curves were analyzed by least-squares interactive convolution method. The quality of the fits was estimated from the goodness-of-fit parameter (χ^2), the residuals, and the autocorrelation function of the residuals.

Transmission electron microscopy (TEM) measurements were performed on a Siemens Elmiskop 101 electron microscope operating at an acceleration voltage of 80 kV. For the observation of aggregates, a drop of sample suspension was placed on 300-mesh formvar copper grids coated with carbon. About 2 min after the deposition, the grid was tapped with filter paper to remove surface water. Staining was performed by addition of a drop of aqueous uranyl acetate solution (0.5%) onto the copper grid. After 2 min, the surface water on the grid was removed by tapping with filter paper.

The samples for cryo-TEM were prepared at room temperature by placing a droplet (5 μL) of the co-assembly PBI solution in water on a hydrophilized (120 s Argon plasma treatment at 70 W using a PlasmaFlecto 10 device of plasma technology GmbH, Germany) perforated carbon-filmed grid (Quantifoil, Germany). The excess fluid was blotted off to create an ultrathin layer (typical thickness of 100 nm) of the solution spanning the holes of the carbon film. The grids were immediately vitrified in liquid ethane at its freezing point (–184 °C) using a standard plunging device (CryoPlunge 3, Gatan, USA). Ultra-fast cooling is necessary for an artifact-free thermal fixation (vitrification) of the aqueous solution avoiding crystallization of the solvent or rearrangement of the assemblies. The vitrified samples were transferred under liquid nitrogen to a FEI Titan 80-300 transmission electron microscope (FEI, Netherlands) using a cryo-holder (Model 626, Gatan, USA). TEM measurements were performed on a FEI Titan 80-300 electron microscope, operating at an acceleration voltage of 300 kV.

Atomic force microscope (AFM). AFM measurements were performed under ambient conditions using a Bruker AXS Multimode Nanoscope IV system operating in tapping mode in air. Silica cantilevers (OMCL-AC160TS) with a resonance frequency of ~300 kHz were used. The samples in aqueous solutions were spin-coated onto Mica under 7000 rpm.

3. Synthesis and characterization of PBI 2

Synthesis of PBI 2. 1,6,7,12-Tetra(4-*tert*-butylphenoxy)perylene-3,4:9,10-tetracarboxylic acid bisanhydride (28 mg, 28 μmol), 3,4,5-tris(3-{2-[2-(ethoxy) ethoxy]ethoxy}-1-propyl)-aniline (40 mg, 65 μmol) and zinc acetate (21 mg, 0.11 mmol) were mixed with imidazole (1 g) and the mixture was stirred at 100 °C for 7 h under argon. After bein cooled down to room temperature, the mixture was dissolved in dichloromethane and extracted with 2*N* HCl (3x) and with aqueous $NaHCO_3$ and dried over $MgSO_4$. After remove of the solvent the crude product was purified by silica gel column chromatography

(dichloromethane/methanol = 97/3 as eluent) to obtain 48 mg (22 μmol, 79%) of PBI **2** as a red solid.

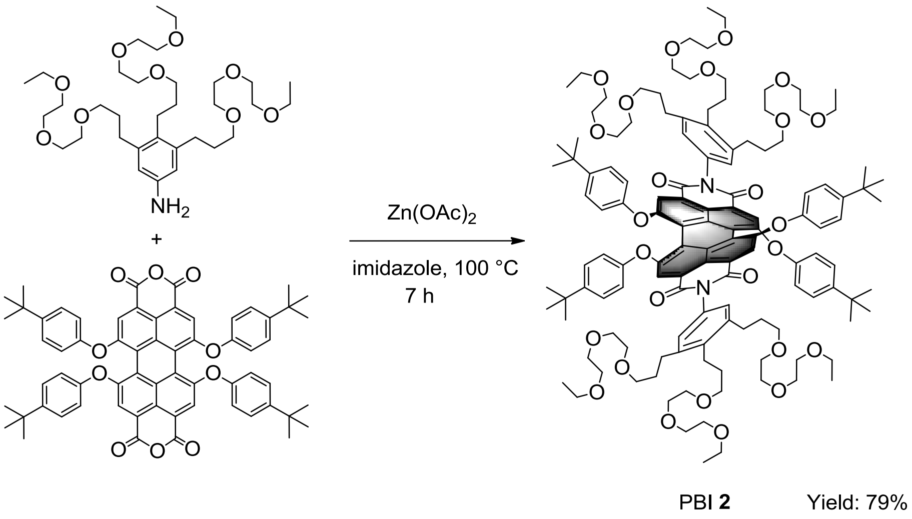

PBI 2: **¹H NMR** (400 MHz, $CDCl_3$, 300 K, TMS) (see **Supplementary Figure 1**, bottom): δ = 8.22 (s, 4H, ArH in perylene ring), 7.22 (m, 8H, ArH), 6.88 (s, 4H, ArH), 6.85 (m, 8H, ArH), 3.68-3.46 (m, 72H, 32x $-OCH_2-$), 2.70 (m, 12H, 6x $ArCH_2-$), 1.85 (m, 8H, 4x $ArCH_2C\mathit{H}_2-$), 1.77 (m, 4H, 2x $ArCH_2C\mathit{H}_2-$), 1.26 (s, 36H, 12x CH_3), 1.20 (t, J = 6.96 Hz, 6H, 2x CH_3), 1.17 (t, J = 7.00 Hz, 12H, 4x CH_3).

¹³C NMR (100 MHz, $CDCl_3$, 300 K, TMS) (see **Supplementary Figure 1**, top): δ = 163.56 (C1, -CO-), 156.04 (C2 in perylene ring), 152.83 (C3 in phenyl rings), 147.3 (C4 in phenyl rings), 141.33 (C5 in perylene ring), 138.55 (C6 in phenyl rings), 133.06 (C7 in perylene ring), 132.58 (C8 in perylene ring), 126.67 (C9 in phenyl rings), 126.59 (C10 in phenyl rings), 122.67 (C11 in perylene ring), 120.63 (C12 in perylene ring), 120.06 (C13 in phenyl rings), 119.72 (C14 in phenyl rings), 119.35 (C15 in phenyl rings), 71.07 (C16, $-OCH_2-$), 70.73 (C17, $-OCH_2-$), 70.66 (C18, C19, $-OCH_2-$), 70.59 (C20,$-OCH_2-$), 70.50 (C21, $-OCH_2-$), 70.22 (C22, $-OCH_2-$), 70.04 (C23, $-OCH_2-$), 69.83 (C24, $-OCH_2-$), 69.80 (C25, $-OCH_2-$), 66.59 (C26, $-\mathit{C}H_2CH_3$), 66.55 (C27, $-\mathit{C}H_2CH_3$), 34.31 (C34, $-\mathit{C}(CH_3)_3$), 31.39 (C35, -

$C(CH_3)_3$), 30.90 (C28, -CH_2CH_2Ph-), 30.68 (C29, -CH_2CH_2Ph-), 29.15 (C30, -CH_2-Ph-), 24.94 (C31,-CH_2-Ph-), 15.14 (C32, -CH_2CH_3), 15.12 (C33, -CH_2CH_3).

MS: (MALDI-TOF, matrix: dithranol) calculated for $C_{130}H_{174}N_2O_{26}$: 2180.77; found: 2181.23 [M+ H^+].

IR ṽ (cm^{-1}): 2955.3, 2925.5, 2861.8, 1738.5, 1762.8, 1668.1, 1585.2,1504.2, 1463.7, 1406.8, 1340.3, 1289.2, 1211.1, 1175.4, 1111.8, 1015.3, 881.3, 839.8, 819.6, 801.3.

UV-vis (dichloromethane) : λ_{ab} (ε, $M^{-1}cm^{-1}$) = 580 nm (4.70 x 10^4), 538 nm (2.91 x 10^4), 451 nm (1.69 x 10^4).

Fluorescence (λ_{ex}= 450nm): λ_{em}= 612 nm (dichloromethane), 596 nm (THF). Fluorescence quantum yield (Φ): 0.97 (THF); fluorescence lifetime (τ = 5.5 ns in THF).

Elemental analysis: calcd (%) for $C_{130}H_{174}N_2O_{26}$ (2180.77): C 71.60, H 8.04, N 1.28; found: C 71.55, H 8.17, N 1.29.

SUPPLEMENTARY REFERENCES

1. Martin, R. B. Comparisons of indefinite self-association models. *Chem. Rev.* **96**, 3043-3064 (1996).

2. Würthner, F., Thalacker, C. & Sautter, A. Hierarchical organization of functional perylene chromophores to mesoscopic superstructures by hydrogen bonding and π-π interactions. *Adv. Mater.* **11**, 754-758 (1999).

3. Zhang, X., Rehm, S., Safont-Sempere, M. M. & Würthner, F. Vesicular perylene dye nanocapsules as supramolecular fluorescent pH sensor systems. *Nature Chem.* **1**, 623-629 (2009).

4. Spiess, H. W. Interplay of structure and dynamics in macromolecular and supramolecular systems. *Macromolecules* **43**, 5479-5491 (2010).

5. Fixler, D., Namer, Y., Yishay, Y., Deutsch, M. Influence of fluorescence anisotropy on fluorescence intensity and lifetime measurement: Theory, simulations and experiments. *IEEE Transactions on Biomedical Engineering* **53**, 1141-1152 (2006).

6. Lackowicz, J. R. Principles of Fluorescence Spectroscopy. 2nd ed. (Kluwer Academic/ Plenum Publishers, New York 1999).

7. Gvishi, R., Reisfeld, R. & Burshtein, Z. Spectroscopy and laser action of the "red perylimide dye" in various solvents. *Chem. Phys. Lett.* **213**, 338-344 (1993).
